# Evaluation of an integrated stepwise rehabilitation model following Nuss procedure in pediatric patients

**DOI:** 10.3389/fped.2025.1591331

**Published:** 2025-09-11

**Authors:** Wen-Jing Li, Li-Li Cao, Chan Li, Jing-Jing Yang, Hui Wang, Yao Tong

**Affiliations:** Department of Anesthesia Surgery, The Second Affiliated Hospital of Air Force Medical University, Xi'an, China

**Keywords:** integrated stepwise rehabilitation training model, nursing model, Nuss procedure, pectus excavatum, pulmonary function

## Abstract

**Objective:**

This study aimed to evaluate the application and rehabilitation outcomes of an integrated stepwise rehabilitation training model in pediatric patients undergoing minimally invasive repair of pectus excavatum (Nuss procedure).

**Methods:**

A cohort of 97 children who underwent the Nuss procedure between July 2019 and August 2021 were included in the study. Participants were divided into an observation group (*n* = 52) and a control group (*n* = 45). The observation group received a stepwise rehabilitation training model in addition to standard perioperative care, while the control group received only routine care. Rehabilitation outcomes, including physical development, pulmonary function, self-care ability, and treatment satisfaction, were assessed over a 12-month period.

**Results:**

There were no statistically significant differences in baseline characteristics or postoperative complications between the two groups. In the observation group, all pulmonary function parameters demonstrated significant improvements at both 6- and 12-months post-surgery (*p* < 0.001). The control group did not indicate significant changes at 6 months, with improvements noted only at 12 months (*p* < 0.001). Postoperative satisfaction and self-care ability in the control group improved significantly at the 12-month follow-up. In contrast, the observation group exhibited significant improvements in these parameters as early as 6 months postoperatively.

**Conclusion:**

Early implementation of an integrated stepwise rehabilitation training model enhances the recovery process in pediatric patients following the Nuss procedure. This approach is both effective and economically feasible, providing a practical framework for post-surgical rehabilitation.

## 1 Introduction

Pectus excavatum, also referred to as funnel chest, is a common congenital deformity of the pediatric chest wall. It is characterized by a funnel-shaped depression involving the sternum, costal cartilage, and sections of the ribs, which curve inward toward the spine. Visible clinical features include a sunken anterior chest, forward-protruding shoulders, mild kyphosis, and a protruding upper abdomen. The deepest point of the deformity typically occurs at the junction of the body of the sternum and the xiphoid process, with severe cases presenting an indentation that approaches the spine (1, 2).

Minimally invasive repair of pectus excavatum (MIRPE) was first introduced by Dr. Donald Nuss in 1998, representing a significant advancement in the treatment of this disease. Internationally, this procedure is commonly known as the “Nuss” procedure (3, 4). Currently, the Nuss procedure is regarded as the preferred treatment approach for pectus excavatum (5, 6). Children affected by this condition are frequently underweight, and present with recurrent upper respiratory infections and restricted physical activity. Post-discharge, the responsibility for their care primarily rests on their caregivers. The capacity of the caregivers to manage postoperative care significantly influences the recovery trajectory and quality of life of the child (7).

Insufficient knowledge regarding rehabilitation exercises among caregivers often impedes recovery. Inadequate guidance on proper rehabilitation may result in complications such as mild kyphosis, abdominal protrusion, abnormal posture, and poor pulmonary function due to the lack of appropriate chest-breathing exercises (8, 9).

In recent years, an integrated stepwise rehabilitation training model has gained prominence as a clinical intervention aimed at enhancing patient prognosis and restoring physical functions. This approach involves the provision of individualized, progressive rehabilitation training tailored to the patient's condition. The model transitions from passive physical exercises to active engagement, with gradually increasing complexity and intensity. Nursing staff play a crucial role in guiding and supervising this process to ensure a stepwise progression (10–12).

The core principle of this rehabilitation model is active patient participation and self-care, facilitating the restoration of coordination between muscle tissue and the nervous system, thereby promoting the recovery of physical functions. To evaluate the efficacy of this integrated stepwise rehabilitation model in the postoperative recovery of pediatric patients undergoing the Nuss procedure, a group of children who underwent the procedure at the Second Affiliated Hospital of Air Force Military Medical University between July 2019 and August 2021 were examined. The children were grouped based on different postoperative rehabilitation models, and the study conducted a detailed assessment of the value of the model in enhancing recovery outcomes following the Nuss procedure.

## 2 Participants and methods

### 2.1 Study participants

This was a retrospective study enrolling 97 pediatric patients who underwent the Nuss procedure between July 2019 and August 2021 were enrolled, with their primary caregivers contributing to patient evaluation and care throughout the study. The participants were divided into an observation group (*n* = 52) and a control group (*n* = 45) based on the year of admission and variations in the nursing model. All surgical procedures were conducted without thoracoscopic assistance, using a 1# wire to secure the steel bar and absorbable sutures for skin closure.

The inclusion criteria for the study were as follows: 1. children aged 3–12 years, 2. children who underwent the minimally invasive Nuss procedure with immediate postoperative removal of the chest tube, and 3. primary caregivers with a minimum of primary school education, the ability to operate a smartphone, and good physical health. Exclusion criteria were: 1. the presence of severe cardiopulmonary diseases in the children or the performance of simultaneous surgeries requiring the insertion of a closed thoracic drainage tube, 2. a history of other trauma or surgeries, 3. postural abnormalities unrelated to pectus excavatum, and 4. non-compliant primary caregivers or those who did not complete follow-up.

Our center adopted an extrapleural lifting technique without thoracic cavity entry, and no thoracic drainage tube was placed postoperatively. A single steel plate was placed in all cases, and the placement method was parallel positioning. Routine collection of complications during hospitalization included pneumothorax, pleural effusion, pneumonia, wound infection, steel bar displacement, sternal fracture, postoperative bleeding requiring transfusion, and reoperation rate. Data were collected by reviewing medical records, imaging reports, and nursing records. All nursing follow-ups were conducted at 6 months and 1 year postoperatively, while medical follow-ups for recurrence were performed annually until the age of 18. The steel bar was removed 1–2 and a half years after surgery considering that early removal may increase the risk of recurrence.

The study was approved by the ethics committee of the hospital, with informed consent obtained from all participants with consent forms signed accordingly. Informed consent was obtained from all participants.

### 2.2 Study methods

#### 2.2.1 Development and specific implementation steps of the integrated stepwise rehabilitation training model

##### 2.2.1.1 Establishment of the post-Nuss procedure rehabilitation training team

The rehabilitation training team was led by the head nurse of the department and comprised of one thoracic surgeon, one rehabilitation physician, and six nurses. The head nurse oversaw the overall progress and optimization of the rehabilitation training plan. The thoracic surgeon conducted follow-up evaluations at various stages of the recovery process, while the rehabilitation physician formulated training plans tailored to the individual conditions of each child and addressed questions that arose during the training sessions. The six nurses, all holding bachelor's degrees with at least a supervisor nurse title, possess specialized knowledge, problem-solving abilities, effective communication skills, and a caring and responsible attitude. Their role involved monitoring and supporting the entire follow-up process.

All medical and nursing staff involved in the rehabilitation training team underwent standardized professional training and were tasked with providing online guidance, follow-up, and the management of training videos after the pediatric patient's discharge. Before discharge, a rehabilitation training manual was prepared, and the primary caregiver was informed of the training objectives and key points to enhance compliance. The manual included comprehensive details such as the names of the pediatric patient and caregiver, sex, age, contact information, the preoperative severity and location of the chest depression, the presence of other diseases, posture and activity levels, surgical method, discharge date, follow-up schedule and so on.

A dedicated WeChat group (a popular communication app in China), titled “Post-Nuss procedure Rehabilitation Training,” was created to disseminate common rehabilitation knowledge and facilitate experience-sharing among caregivers. Guardians were allowed to apply for group membership voluntarily, with approval from the group administrator, and all members could see each other's messages. Prior to joining, all guardians were fully informed and provided voluntary consent to participate in the group.

Additionally, smaller groups were formed for caregivers of children who underwent surgery in the same month, enabling targeted guidance for children at similar stages of rehabilitation.

##### 2.2.1.2 Development of the integrated stepwise rehabilitation training plan

The rehabilitation plan emphasized the synchronized training of both respiratory and motor functions, both of which were crucial for recovery. The first component focuses on respiratory function rehabilitation exercises. Proper breathing techniques were used to enhance lung capacity, thereby establishing a foundation for subsequent physical rehabilitation. Research indicates that postoperative complications following the Nuss procedure can occur in up to 15.2% of cases, with common complications including pneumothorax, atelectasis, pneumonia, and pleural effusion (2).

Postoperative discomfort, such as pain and the sensation of foreign objects in the chest wall, often leads to mouth breathing and the avoidance of coughing, which can hinder pulmonary function. To address this issue, nurses guided patients through diaphragmatic breathing exercises. These exercises were initially performed while the pediatric patient was awake and lying flat and were later incorporated into the broader exercise routine as part of the rehabilitation program.

The second component of the rehabilitation plan involved motor function exercises, which were divided into four progressive stages designed to match the physical capacity of the pediatric patient.

Stage 1: Adaptive training aims to help the pediatric patient gradually adjust to the presence of the implanted steel plate and regain normal walking, without emphasizing walking speed.

1. Within 6 h post-surgery: Once the pediatric patient was fully awake and stable, a pillow was provided, and the head of the bed was elevated to 30°. Based on their condition and pain tolerance, the elevation was increased incrementally by 15° each hour, with the goal of achieving an upright sitting position at a 90° angle within 12 h. Within 24 h post-surgery: The urinary catheter was removed, and the pediatric patient was assisted in getting out of bed to use the toilet. During the first three postoperative days, the focus was placed on exercises involving lying down and sitting up. Family members were encouraged to provide support by applying gentle pressure to the back of the pediatric patient and assisting with the bed rail to help them sit upright. Instructions were given to maintain proper posture, including keeping the shoulders horizontal, the head and chest elevated, and the waist aligned vertically with the bed.
2. Post-24 h: The pediatric patient may begin walking and practice standing against a wall. Adaptation to lying flat for sleep and conscious correction of habitual gait errors were emphasized in the first three days. Throughout these exercises, care was taken to prevent breath-holding or hyperventilation due to pain. The pediatric patient was guided to use correct breathing techniques during physical activities to support recovery.

Stage 2: Restorative Self-Care Training focuses on rehabilitation exercises during the first month after surgery.

1. Postoperative Days 10–20: Daily exercises included alternating between wall-standing and upright sitting postures. Each session lasted 5 min and was performed three times per day. For pediatric patients with preoperative kyphosis, the frequency was increased to 5–6 sessions per day. Walking exercises were continued with the use of a pedometer to ensure a minimum of 1,000 steps daily. Gradually, caregivers reduced their level of assistance with the movements of the pediatric patient in and out of bed.
2. Postoperative Days 21–30: Upper limb exercises emphasized raising, abduction, and internal rotation, while lower limb exercises focused on squatting and lunges to strengthen core muscles and improve lower limb stability. These exercises help build balance for future activities and reduce the risk of falls during physical exertion. By this stage, pediatric patients were expected to independently perform basic activities such as dressing and maintaining personal hygiene.

If signs of reduced endurance, shortness of breath, or minor pleural effusion were detected during follow-up evaluations, a “dual-purpose breathing trainer” may be introduced to support lung capacity improvement.

Stage 3: Strengthening Training typically begins between 30- and 60-days post-surgery. During this phase, the pediatric patient began by lying on their back with a 10 cm soft pillow, gradually transitioning to a side-sleeping position as tolerated. The focus is on strengthening the upper limbs through exercises such as chest expansion, arm swings with backward kicks, and clasping the hands behind the back. These exercises were performed in sets lasting 10 min each, with three sets per day. Proper posture was emphasized to prevent chest rounding and chin tucking during exercises.

Stage 4: Training Consolidation begins between 61- and 90-days post-surgery. This stage builds on the exercises from the strengthening phase by incorporating additional movements, such as trunk rotations and simple jumping exercises, including knee raises that touch either the same-side or opposite elbow. Preliminary sports activities such as jump rope, badminton, and table tennis were introduced based on the progress of the pediatric patient. However, high-impact activities like basketball and football were avoided during this period.

Avoiding the rushing of the rehabilitation process was crucial throughout this period. The duration and intensity of exercises were adjusted according to the physical capabilities of the pediatric patient, and the stage was extended if necessary. If symptoms such as worsening pain occurred and were not alleviated, rehabilitation training should be discontinued immediately, and medical consultation should be sought.

#### 2.2.2 Postoperative care and discharge guidance

The control group did not receive any special postoperative care. They only received standard postoperative care, including routine discharge instructions. In contrast, the observation group began nursing interventions under the integrated stepwise rehabilitation training model beginning six hours post-surgery. Both the pediatric patient and the primary caregiver were instructed to adhere strictly to the daily rehabilitation training plan. A dedicated WeChat group was created for regular check-ins, where designated staff monitored progress and ensured that the duration and quality of exercises met established requirements.

For analgesia management, local infiltration with 0.25 mg/ml ropivacaine hydrochloride injection was administered around the bilateral incisions for patients aged 6 years and above. Additionally, patient-controlled analgesia (PCA) was configured by diluting 2 μg/kg sufentanil citrate injection and 5 mg tropisetron to 100 ml, with a pump speed set at 1.5 ml/h. In contrast, an analgesic pump was not routinely administered to patients aged 3–6 years; only local infiltration anesthesia was applied around the incision. Oral antipyretic analgesics were administered postoperatively based on the degree of pain.

### 2.3 Observation indicators

• Lung Function Measurements: Lung function indicators were assessed at various time points using the blowing method with a traditional spirometer. This procedure involved instructing the pediatric patient to take a deep breath and exhale fully into a measuring container until no further air could be expelled. The lung volume was determined by reading the measurement from the scale on the container.
• Activities of Daily Living (ADL) Assessment: The ability of the pediatric patient to perform daily activities was evaluated using the Barthel Index scoring scale. The level of self-care was categorized, and patient cooperation was classified as either partial or complete self-care based on the total score.
• Satisfaction Assessment: Caregiver satisfaction was assessed through a survey utilizing a Likert scale and a binary classification method to categorize responses.

### 2.4 Statistical methods

Data analysis was conducted using SPSS version 22.0. Continuous variables following a normal distribution were presented as mean ± standard deviation $\left(\bar{x}\pm s\right)$, and differences between groups were assessed using independent-sample *t*-tests. Categorical variables were expressed as frequencies and percentages (*n*%), with differences analyzed through Fisher's exact test. A two-sided significance level was applied, and a *p*-value of <0.05 was considered statistically significant.

## 3 Results

### 3.1 Comparison of general data

A total of 97 pediatric patients who met the inclusion criteria were enrolled in the study. Participants were divided into an observation group and a control group based on the principle of maintaining comparable baseline characteristics. The observation group comprised of 52 patients (38 boys and 14 girls), with a median age of 6.70 ± 3.83 years. The control group consisted of 45 patients (31 boys and 14 girls), with a median age of 6.58 ± 3.97 years. No statistically significant differences were observed between the two groups in terms of general characteristics, including sex, age, Haller index, and classification (*p* > 0.05), confirming the comparability of the groups (Table 1).

**Table 1:** General and perioperative conditions of the two pediatric patient groups.

Item	Control group (*n* = 45)	Observation group (*n* = 52)	Statistic (*t*/*χ*^2^)	*P*-value
Age/Years	6.58 ± 3.97	6.70 ± 3.83	0.916	0. 25
Sex			2.206	0.650
Male	31 (68.9%)	38 (73.1%)		
Female	14 (31.1%)	14 (26.9)		
Haller index	5.3 ± 4.7	4.9 ± 2.0	0.801	0.424
Recurrence cases	4 (8.9%)	8 (15.4%)	0.939	0.333
Classification			7.786	0.675
Symmetrical	32 (71.1%)	41 (78.8%)		
Asymmetrical	8 (17.8%)	7 (13.5%)		
Unbalanced	5 (11.1%)	4 (7.7%)		
Surgery time	48.63 ± 12.66	50.99 ± 17.24	3.406	0.714
Intraoperative blood loss	6.2 ± 1.3	5.9 ± 1.5	5.213	0.11
Postoperative hospital stay	1.6 ± 0.5	1.7 ± 0.4	4.357	0.12
Postoperative complications	1 (2.2%)	2 (3.8%)	2.212	0.645

### 3.2 Comparison of postoperative follow-up of lung volume, activities of daily living, and satisfaction

The comparison of lung function indicators in pediatric patients with pectus excavatum was conducted at three time points: before surgery, at 6 months postoperatively, and at 12 months postoperatively. In the control group, no significant changes in lung function indicators were observed at 6 months post-surgery compared to preoperative values (*p* > 0.05). However, by 12 months postoperatively, both FVC and FEV1 had significantly decreased compared to preoperative levels (*p* < 0.001). Conversely, forced expiratory flow at 50% and 75% of vital capacity (FEF50% and FEF75%) indicated significant increases (*p* < 0.001) (Table 2).

**Table 2:** Comparison of the lung function of the control group prior to and following surgery $\left(\bar{x}\pm s\right)$.

Control group					
Pulmonary function indicator	Pre-surgery	6 months post-surgery	12 months post-surgery	*P*-value^a^	*P*-value^b^
FVC	80.6 ± 14.5	78.1 ± 13.9	70.3 ± 14.6	0.302	<0.001
FEV1	81.2 ± 15.6	78.8 ± 15.9	74.1 ± 14.6	0.379	<0.001
FEF25%–75%	84.4 ± 25.1	82.4 ± 24.8	97.5 ± 9.4	0.695	0.120
FEF50%	82.6 ± 27.0	85.7 ± 27.5	91.8 ± 27.9	0.588	0.075
FEF75%	77.7 ± 33.7	80.5 ± 31.2	118.9 ± 45.3	0.686	<0.001

^a^ Comparison of 6 months post-surgery with pre-surgery.

^b^ Comparison of 12 months post-surgery with pre-surgery.

In the observation group, FVC and FEV1 were significantly reduced at both 6 and 12 months postoperatively compared to preoperative values (*p* < 0.001). FEF50% and FEF75% demonstrated significant increases at both time points (*p* < 0.001), while forced expiratory flow between 25% and 75% of vital capacity (FEF25%−75%) indicated no statistically significant difference (*p* = 0.328) (Table 3).

**Table 3:** Comparison of the lung function prior to and following surgery in the observation group $\left(\bar{x}\pm s\right)$.

Observation group					
Pulmonary function indicator	Pre-surgery	6 months post-surgery	12 months post-surgery	*P*-value^a^	*P*-value^b^
FVC	79.9 ± 14.3	71.3 ± 14.5	72.0 ± 14.1	<0.001	<0.001
FEV1	82.4 ± 14.0	75.6 ± 14.7	77.3 ± 15.0	<0.001	<0.001
FEF25%–75%	89.4 ± 23.6	93.3 ± 23.0	95.6 ± 27.2	0.328	0.022
FEF50%	84.1 ± 22.3	93.5 ± 39.4	90.8 ± 23.7	0.007	0.006
FEF75%	91.3 ± 33.4	106.7 ± 32.2	107.3 ± 35.7	<0.001	<0.001

^a^ Comparison of 6 months post-surgery with pre-surgery.

^b^ Comparison of 12 months post-surgery with pre-surgery.

No serious or life-threatening complications occurred in either group during the perioperative period. The prognosis remained favorable throughout the 6-month to 1-year postoperative follow-up. No statistically significant difference in satisfaction was found between the groups at 6 months postoperatively (*χ*^2^ = 2.933, *p* = 0.087). However, by 12 months, satisfaction levels were significantly higher in the observation group compared to the control group (*χ*^2^ = 3.929, *p* < 0.05), with both patients and their caregivers expressing increased satisfaction (Table 4).

**Table 4:** Postoperative satisfaction survey of both groups.

Grouping	6 months post-surgery		12 months post-surgery	
	Satisfied	Dissatisfied	Satisfied	Dissatisfied
Control group	35	10	38	7
Observation group	47	5	50	2
Statistic (*χ*^2^)	2.933		3.929	
*P*-value	0.087		0.047	

Postoperative self-care abilities were assessed at 6 months and 12 months post-surgery. At 6 months, no significant difference in self-care ability was observed between the groups (*p* > 0.05). By 12 months, a statistically significant improvement in self-care ability was evident in the observation group, where a higher proportion of pediatric patients demonstrated complete self-care compared to the control group (*p* < 0.05) (Table 5).

**Table 5:** Postoperative self-care ability survey of both groups.

Grouping	Partially self-care	Completely self-care	Partially self-care	Completely self-care
Control group	17	28	6	39
Observation group	17	35	1	51
Statistic (χ^2^)	0.274		4.574	
*P*-value	0.601		0.032	

## 4 Discussion

### 4.1 Integrated stepwise rehabilitation training model corrects pre-existing and post-operative postural abnormalities

Children with pectus excavatum often present with a slender body type, forward-shifted shoulders, and in some cases, scoliosis. Although the Nuss procedure corrects the depressed sternum, chronic poor posture does not resolve immediately postoperatively. Long-term, systematic, and professionally guided rehabilitation is required for postural improvement (13).

In this study, both groups experienced some degree of postoperative hunching due to discomfort from the implanted steel plates, with one case in the control group and two cases in the observation group. Pediatric patients in the observation group demonstrated quicker correction of hunching and slouched shoulders through structured rehabilitation training. Postoperatively, pediatric patients exhibit a forward-bending posture as a response to pain, and caregivers assume this issue will resolve naturally as pain decreases. However, poor posture hinders the full corrective effect of the steel plate, contributing to improper sitting and walking habits that become difficult to correct over time. These postural issues have long-term consequences on physical growth, development, and psychological well-being.

The integrated stepwise rehabilitation training model implemented in this study facilitates adaptation to the friction caused by the steel plates, promoting the maintenance of an upright posture and supporting optimal recovery.

### 4.2 Integrated stepwise rehabilitation training model increases lung capacity and enhances pulmonary function

Children with pectus excavatum often exhibit impaired cardiopulmonary function, which increases the risk of respiratory conditions such as pneumonia and bronchopneumonia (14). Symptoms such as shortness of breath and reduced endurance during daily activities may be present prior to surgery. Postoperatively, pediatric patients frequently adopt mouth breathing due to pain at the incision site, discomfort from the implanted steel plate, and the sensation of a foreign body during respiration (15).

Targeted respiratory rehabilitation training, including the use of breathing training devices when necessary, improves lung capacity and mitigates or even resolves symptoms of shortness of breath during physical activities. Postoperative respiratory exercises decreases the risk of complications such as difficulty in expectoration, atelectasis, and pleural effusion (16). Consistent respiratory and physical exercise contributes to the normal development of the thorax and helps prevent suboptimal outcomes after the removal of the steel plate.

In this study, both groups demonstrated improved lung function at 12 months postoperatively compared to preoperative levels. However, the observation group, which participated in the integrated stepwise rehabilitation training model, experienced faster pulmonary recovery in the early postoperative period than the control group. This approach not only facilitated the early resumption of normal daily activities and academic participation but also supports its broader clinical application. The mean age of the enrolled patients was approximately 6 years. This could be attributed to two primary factors. First, our center serves as the largest diagnostic and treatment facility for chest diseases in Western China, which attracts a substantial number of patients—including preschool-aged children—seeking medical care. Second, the disease demonstrates a higher likelihood of self-detection, early diagnosis, and prompt treatment at younger ages. The medical team at our center has observed that early intervention for children aged 3–6 years yields remarkable efficacy, with the potential to achieve full recovery to normal physiological levels within a one-year period.

### 4.3 Integrated stepwise rehabilitation training model helps pediatric patients achieve independent living skills earlier

Primary caregivers often lack the specialized knowledge necessary for effective postoperative rehabilitation, making it challenging for them to recognize and correct poor habits in their children. This knowledge gap contributes to heightened anxiety regarding pain management and increased family stress. The integrated stepwise rehabilitation training model addresses this issue by incorporating common postural habits into each stage of rehabilitation. Pediatric patients are guided through exercises designed to progressively enhance their ability to perform daily activities independently under the supervision of the medical team.

This structured approach accelerates the rehabilitation process, enabling them to achieve full independence more quickly and improving overall treatment satisfaction. In this study, self-care abilities were assessed using the Barthel Index. The observation group reached mild dependency standards in a shorter timeframe than the control group.

## 5 Conclusion

The integrated stepwise rehabilitation training model developed in this study effectively corrects poor postural habits, facilitates the rapid recovery of self-care abilities, enhances cardiopulmonary function, and accelerates overall rehabilitation, all while maintaining patient safety. This model supports early reintegration into normal activities, including daycare and school attendance. However, certain limitations remain, particularly in the areas of pain management and psychological care for children. Further research is required to develop a more comprehensive and integrated postoperative care model.

It is essential for healthcare professionals to receive specialized training in rehabilitation to enhance their ability to identify postoperative recovery issues promptly. Strengthening communication with primary caregivers is equally important to continuously refine the rehabilitation plan and address emerging needs.

This study is subject to several limitations. It was conducted as a single-center retrospective study with a relatively small sample size, which may have introduced selection bias. Additionally, the lack of random group allocation underscores the need for further in-depth research using larger, multi-center, randomized designs to improve the generalizability of the findings.

Despite these limitations, the findings of the study indicate that with clearer intervention protocols and improved observation indicators, the integrated stepwise rehabilitation model holds significant potential for clinical application. Its refinement could help address market needs and facilitate the successful translation of research findings.

## Data Availability

The original contributions presented in the study are included in the article/Supplementary Material, further inquiries can be directed to the corresponding author.
